# Acute postoperative paracentral corneal melting and perforation after uncomplicated cataract surgery in a patient with inadvertent rheumatoid arthritis

**DOI:** 10.5935/0004-2749.202100108

**Published:** 2021

**Authors:** Alejandro Rodriguez-Garcia, Andres Bustamante-Arias, Julio C. Hernandez-Camarena, Denise Loya-Garcia, J. Carlos Alvarez-Guzman, Luis A. Rodriguez-Gutierrez

**Affiliations:** 1 Tecnologico de Monterrey, School of Medicine and Health Sciences, Institute of Ophthalmology and Visual Sciences, Monterrey, Mexico

**Figure 1:**
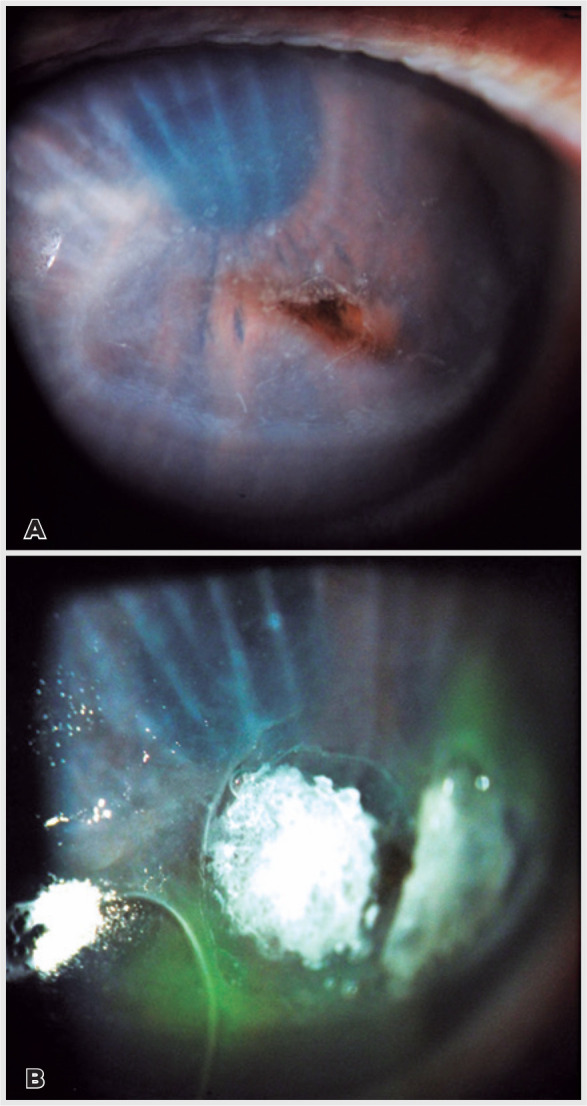
(A) Left cornea with a paracentral fusiform perforation, flat anterior chamber with iris-endothelium touch, oval stromal infiltrate with edema, and prominent radial Descemet folds. (B) Transient cyanoacrylate glue corneal tamponade and bandage contact lens.

**Figure 2:**
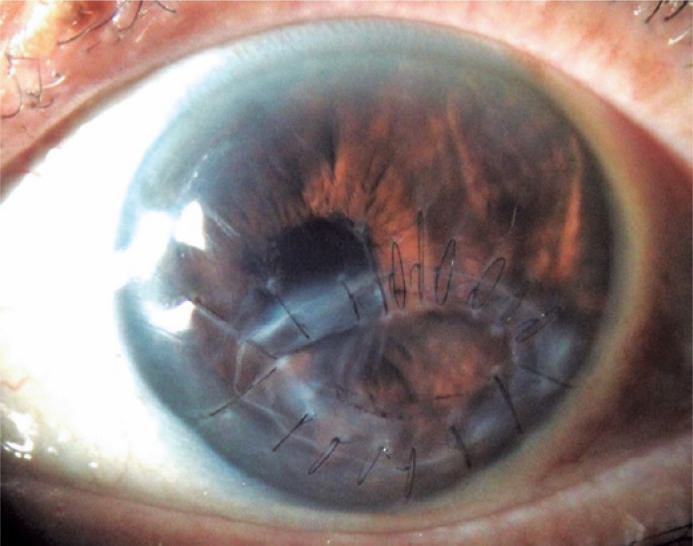
Clear and compact oval corneal tectonic graft with formed anterior chamber and slight corectopia.

Dear Editor,

Acute central or paracentral autoimmune corneal ulceration (CPCU) is a distinct clinical entity to peripheral ulcerative keratitis (PUK) potentially presenting in patients with occult systemic vasculitis and rheumatoid arthritis (RA). A prominent clinical feature distinguishing CPCU from PUK is low-grade inflammatory processes associated with CPCU at initial presentation^(1)^. We recently read the interesting case report of a paracentral corneal stromal melting after uncomplicated phacoemulsification in a patient with undiagnosed RA by Dervenis et al.^(2)^. This reminded us of a 78-year-old Hispanic female who developed an indolent and rapidly progressive paracentral corneal ulceration with perforation six days after an uneventful cataract phacoemulsification. The lesion was characterized by a fusiform-shaped, full-thickness defect (2.5 × 3.5mm) surrounded by a discrete inflammatory oval shaped stromal infiltrate, and 2+ edema with significant Descemet radial folds (Figure 1A). The patient was referred to our clinic for diagnosis and treatment with a CDVA of CF at 2 ft. Topical 0.5% moxifloxacin, 1% atropine, and systemic ciprofloxacin (1g/day) were administered. Immediate anterior chamber reformation and cyanoacrylate glue plus a bandage contact lens were applied to the corneal perforation (Figure 1B). The patient complained of mild hand arthralgias, while no signs or symptoms of dry eye were observed. An extensive laboratory work-up was remarkable for positive rheumatoid factor (30.4 UI/ml) and ANA (1:1280 centromere pattern) with negative anti-DNA, anti-Sm, anti-RNP antibodies, elevated ESR (27 mm/h) and CRP (16.1UI/ml), and a negative ANCA test. A rheumatology consultation subsequently confirmed RA. The patient was treated with oral prednisone (40mg/day) and methotrexate (15mg/week PO) and was submitted for a corneal tectonic graft. Surgery was planned as a 4-5mm diameter round corneal graft to cover the perforation area, however, during necrotic tissue debridement, the entire surrounding stroma at the edge of the oval-shape infiltration was friable and thus removed. The debrided corneal tissue was negative for microorganisms from smear and culture analysis. Seven months after surgery, the patient was still on continuous immunosuppressive therapy and intense preservative-free topical lubricants but had a clear and compact cornea with an improved CDVA of 20/40 (Figure 2).

In terms of the known risk factors for developing this unexpected, sight-threatening complication, but distinct to the case by Dervenis et al.^(2)^, our patient did not have any previously known predisposing factors, such as radiation therapy, which predisposes to neurotrophic keratopathy, significant dry eye disease, or infection at the time of diagnosis. However, as in the referred case, our patient also had undiagnosed RA. Other previous similar cases have shown common predisposing factors, including dry eye disease, autoimmune disease, particularly RA associated with or without Sjögren syndrome and diabetes mellitus, topical corticosteroid and NSAID use, and other related conditions such as radiation therapy and concomitant pterygium excision^(3-5)^. The pathogenic mechanism underlying this rapidly progressive corneal stromal degradation is unclear. Interestingly, cases with sterile central or paracentral ulceration are not clinically associated with a significant stromal inflammatory infiltrate, but still rapidly progress to perforation. There is no accompanying ciliary injection, anterior chamber, or scleral inflammation. Moreover, patients with known RA usually have a quiet systemic disease^(1)^.

From a histopathological perspective, previous studies have variably observed HLA-DR expression on epithelium and stromal keratocytes, pro-inflammatory cytokine, IL-6, TNFα, and collagenase (MMP-1) overexpression at the lesion site, immunoglobulin deposition in the corneal epithelium, subepithelial T-lymphocyte infiltration, stromal neutrophils at the site of ulceration, and ‘an absence of B-lymphocyte reactivity^(1,5)^. Although histopathologically varied, these findings suggest complex adaptative immune responses involving mononuclear cell (T-lymphocytes and macrophages) and neutrophil participation in inflammatory processes^(1,3-5)^. Some studies have compared corneal immunohistopathological CPCU findings to the synovial fluid in RA inflamed joints^(4,5)^. Finally, biochemical and ultra-structural collagen alterations, similar to those in RA have also been observed in the melted stroma^(1,5)^.

In conclusion, CPCU is a rapidly progressive indolent, but sight-threatening complication after uneventful cataract surgery. Suspicion and early recognition of potential risk factors potentially triggering this inflammatory process are crucial to avoid corneal perforation and subsequent consequences. Also, mandatory long-term systemic immunosuppression has a crucial role in avoiding further systemic and ocular complications in cases.
